# Ophthalmia Neonatorum

**Published:** 1888-03

**Authors:** Robert Tilley


### OPHTHALMIA NEONATORUM OR PURULENT OPHTHALMIA OF THE NEW-BORN.

BY ROBERT TILLEY, M. D.

[A Clinical Lecture Delivered at St. Luke's Hospital.]

The technical name employed to designate the affection that I wish to discuss with you this afternoon, gives you no adequate conception of its importance. In order to comprehend its importance it is necessary to try and form an idea of the extent to which it prevails and the ravages which it leaves in its tracks.

Several years ago the question of prophylaxis of this affection was studied by several of the profession, more especially the Germans, and to Professor Credé, whose name is, or certainly will become, familiar to you in the study of obstetrics, belongs the honor of presenting the most efficient method. It was shown that in the general hospital in which he labored, in round numbers one case of ophthalmia neonatorum occurred in ten births—ten per centum. Now, men and women are much the same the world over, and given a population equally dense to the one in which Professor Credé made his observations, we may expect pretty nearly the same results. As a matter of fact, I remember a report several years ago from the Maternity Hospital of New York, in which the number of cases of ophthalmia neonatorum was given as practically the same as that recorded by the German professor—10, 10.5, 11 per centum. This, however, was previous to the adoption of the prophylactic measures worked out by Credé. I shall refer to these measures later.

But you are, perhaps, ready to exclaim, "Yes; but that is in hospital cases, and consequently come from the poorer people." Let me remind you, however, that the affection to which this ophthalmia of the new-born owes its origin, is no respecter of persons, and is by no means confined to the poor. It will certainly make its appearance in your own practice unless you are very much more punctilious and observant than your confrères, and even then you will encounter it in the experience of others. There is, however, no possibility of ascertaining the proportion of cases in which it occurs in private practice.

But whilst it is impossible to ascertain the percentage of cases in private practice, if we study it from another standpoint we shall be convinced that it is not infrequent. Magnus, of Breslau, says that in the blind asylum under his charge, I forget now in what year, thirty-four per centum of all the blind in the asylum were blind from ophthalmia neonatorum. He is careful to add, however, that the inmates of the institution are largely young people, and such a percentage could not exist if we take into consideration the whole of those afflicted with blindness. His percentage of blind, however, from purulent ophthalmia, is not unique at all; a percentage still higher has been recorded both in England and Russia, as well as other countries.

In his excellent work, " Die Blindheit, ihre Entstehung und ihre Verhütung," Dr. Magnus has collated 2,528 cases of total blindness recorded by excellent observers in Germany—Schmidt, Rimpler, Uhthoff, Stolte, Hirschberg, Landesberg, himself and others. He has presented these in graphic form, which I have reproduced for you; and of all the affections causing total blindness, ophthalmia neonatorum takes the precedence in its destructive influence on the eyes, the proportion being 10.87 per centum; that is to say, that out of ten blind persons, one, at least, was blind from purulent ophthalmia of the new-born. History repeats itself, gentlemen, and what is true in Germany will have its counterpart here in America. As a matter of fact, according to the census of America of 1880, out of ten thousand individuals there were 9.73 blind, whilst a carefully made compilation of Germany gives in ten thousand inhabitants only 8.79. Accordingly, in round numbers, we may be said to have in America 50,000, blind; and it would be fair to conclude that ten per cent of this 50,000, or 5,000, are blind from purulent ophthalmia of infancy. This number, however, it must be remembered, takes no account whatever of those who have been afflicted with the disease and have been cured, or simply deformed with leucoma or afflicted with the simple loss of one eye only; it takes cognizance only of those who are incurably blind. There is yet another idea in connection with this class of cases which will not fail to impress you with its importance, namely, the occurrence of blindness at this early age means the total destruction of the most active avenue of knowledge. Blindness at forty to sixty is a dire calamity, but blindness from infancy, who or what can compensate for it?

This disease usually reveals itself from twelve to forty-eight hours after delivery. You will readily see, however, that the rapidity of its manifestation will depend on several conditions. If the labor is a tedious one, and the head remains a long time in the vaginal canal, its manifestation after complete delivery will probably be earlier.

Nor must we forget that in all probability the stage in which the corresponding affection exists in the mother will probably contribute largely to the time of its development as well as to the virulence of its form. One of its first symptoms is photophobia; then redness and swelling of the conjunctiva; serous and purulent discharge. The purulent discharge is frequently very abundant and the swelling sometimes so extensive that the upper lid extends considerably over the edge of the lower one. It is true, the affection in infants is frequently less violent than the corresponding affection in adults. Yet the number of blind referred to this cause shows that it is frequently severe enough in infants. When it results in blindness it is frequently associated with leucoma, or a milk-white condition of the cornea, together with more or less destructive changes in the deeper structures of the eye ; or the anterior part of the eye becomes staphylomatous.

The most important point to determine previous to a prognosis is to ascertain the condition of the cornea. If it is still clear, and complete control of the case can be obtained, there should as a rule be no serious impairment of vision from this source. It must, however, be remembered that cases of congenital cataract or congenital atrophy of the optic nerve, enjoy no immunity whatever from purulent infection, and hence a case of purulent ophthalmia of the new-born when relieved of the ophthalmia, may reveal signs of congenital cataract or optic-nerve atrophy wholly independent of the affection in question. Should the cornea be cloudy when it is first seen the prognosis must always be very guarded, as, at the best, leucoma of the cornea will probably result.

The treatment of these cases varies greatly with different practitioners. There are some who claim that frequent washing of the eyes, so as to cleanse them from the pus, is the all-important item in the treatment. And such do not hesitate to characterize the use of nitrate of silver, so highly prized by others, as barbarous. Others are content with a saturated solution of boric acid for instillation in the eye, after cleansing. Others use solutions of sulphate of zinc and sulphate of aluminum and potassium; others adhere by preference to the nitrate of silver. Of those who use solutions of nitrate of silver, many differ in opinion widely relative to the strength of solution preferred.

Some of the English ophthamologists use by preference the mitigated nitrate of silver sticks; that is, nitrate of silver and nitrate of potassium in various proportions. Personally, I give the preference to a solution of nitrate silver of about 20 to 30 or even 40 per centum of nitrate silver in distilled water. In using these strong solutions, however, great care must be taken to protect the cornea, and any excess of the silver nitrate solution should be immediately washed away. I prefer to apply it with a little cotton-wool on the end of a probe—I do not use a brush. Once in twenty-four hours is sufficently frequent for such applications, but meanwhile the eyes should be kept scrupulously clean. Cleanliness can be secured only by very frequent washing out of the cul-de-sacs. The swelling and discharge diminish greatly in the first twenty-four hours. Immediately after the application there is quite an abundant exudation of serum, and the lids should be opened to allow its escape. The application of a little vaseline to the edges of the lids is serviceable to prevent the edges from adhering to each other. When they are adherent, however. the application of a little warm water soon overcomes the difficulty. I cannot too urgently caution you against the danger of allowing any of the secretion to come in contact with your own eyes, and you in your turn must be equally cautious in warning those who have charge of an infant so afflicted.

The little one that illustrates this affection for you to-day was not afflicted with a very violent form. It evidently was not observed or recognized as such until about the fifth day after delivery. I did not see it until the eighth day. That was about twelve days ago, and whilst the pus rolled out from between the eyelids when I first saw it, there is now little or no extra secretion. There remains, however, still a hyperæmia of the conjunctiva and I will now apply again the nitrate of silver solution. I take my position immediately opposite the nurse; the child is held on its back in her lap and its head between my knees; the lids are readily everted and the superior border of the upper and lower border of the lower tarsal cartilages brought together so as to protect the cornea from the solution of nitrate of silver; 20 per centum solution is applied, allowed to remain a few seconds, and then washed off with water and the lids returned to their positions.

The prophylactic measures which I referred to before, as advocated by Professor Credé, are in substance as follows: The eyelids are washed and carefully wiped as early as possible after delivery. The eyelids are then slightly separated, and a single drop of a two per centum solution of nitrate of silver is made to drop from a glass rod on the cornea and allowed to flow equally into the upper and lower conjunctival cul-de-sacs. This constitutes the whole of the prophylactic measures. This method has justly become so popular that it is taught in the midwifery courses. The application, according to report, gives no pain and no prolonged discomfort. Only in those prematurely born does a slight amount of conjunctivitis ensue, and this difficulty disappears in a very brief period. When we learn that it has resulted in the complete obliteration of ophthalmia neonatorum from the maternity wards and its consequent disastrous results in after life, it must be considered well worthy of attention. Any method which will diminish the number of blind of a country 10 per centum must be a great boon to humanity. Were I in general or obstetric practice, I certainly should adopt the method at any rate in all cases in which the mother gave evidence of suspicious leucorrhœa.

I have said that the application of the two per centum solution of nitrate of silver gave rise to little or no discomfort or evil results. There has, however, recently been a case reported in the New York "Medical Record" by Dr. Pomeroy in which troublesome hæmorrhage is said to have occurred, but the practitioner that Dr. Pomeroy refers to cannot be said to have followed Credé's directions. He is said to have dropped the nitrate of silver solution from a spoon onto the baby's eyes. Now, gentlemen, in the first place, a spoon is not an instrument at all adapted for use with nitrate of silver; in the second place, it is practically impossible to drop from a spoon, especially when it has to be guided into a child's eye; and in the third place, Credé recommends the very best thing for the purpose—a glass rod. Now if you ever adopt the method, do not despise the glass rod, and never attempt to do it with a spoon.

There is one more point to which I would call your attention. Gonorrhœal infection is very generally said to be caused by a minute organism, the gonococcus; and of course the ophthalmia of the new-born must be referred to the same influence. Nitrate of silver ranks very high as a germicide, especially with certain germs. This is very interestingly set forth in a book on "Ferments et Maladies," by Duclaux. After studying the culture-fluid of Ravlin, specially devised for the culture of the aspergillus, he says that if to the ideal solution 1-1,600,000 of nitrate of silver be added the further development ceases at once, and that a similar ideal solution that will give three crops of aspergilli in three days and in doing so exhaust itself of practically all its constituents except the excess of water; if the same kind of solution be placed in a silver vessel the seeds of aspergilli when sown on its surface refuse to develope. It may be that the nitrate of silver, whether used as a prophylactic or as a remedy to cure, acts in a similar way.

